# *QuickStats:* Percentage* of Residential Care Communities^†^ Engaged in Selected End-of-Life and Bereavement Care Practices^§^ — National Study of Long-Term Care Providers, United States, 2018

**DOI:** 10.15585/mmwr.mm7038a7

**Published:** 2021-09-24

**Figure:**
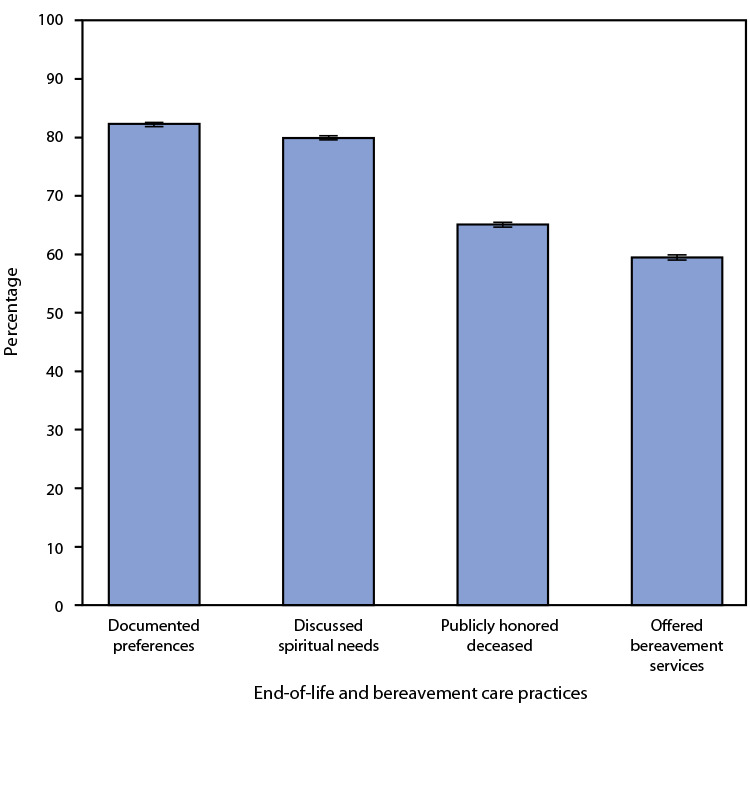
In 2018, when a resident was dying or died, 82% of RCCs documented residents’ family, religious, or cultural preferences in their care plans, 79.9% discussed residents’ spiritual needs with them, 65.1% publicly honored deceased residents in the RCC, and 59.5% offered bereavement services to staff members and residents.

**Source:** National Study of Long-Term Care Providers, 2018. https://www.cdc.gov/nchs/npals/index.htm

For more information about this topic, CDC recommends the following link: https://www.cdc.gov/aging/advancecareplanning.

